# Supplementary figures and images for: Causal inference for heritable phenotypic risk factors using heterogeneous genetic instruments
Source: PLoS Genet. 2021 Jun 22;17(6):e1009575. doi: 10.1371/journal.pgen.1009575 (PMC8301661; doi:10.1371/journal.pgen.1009575)

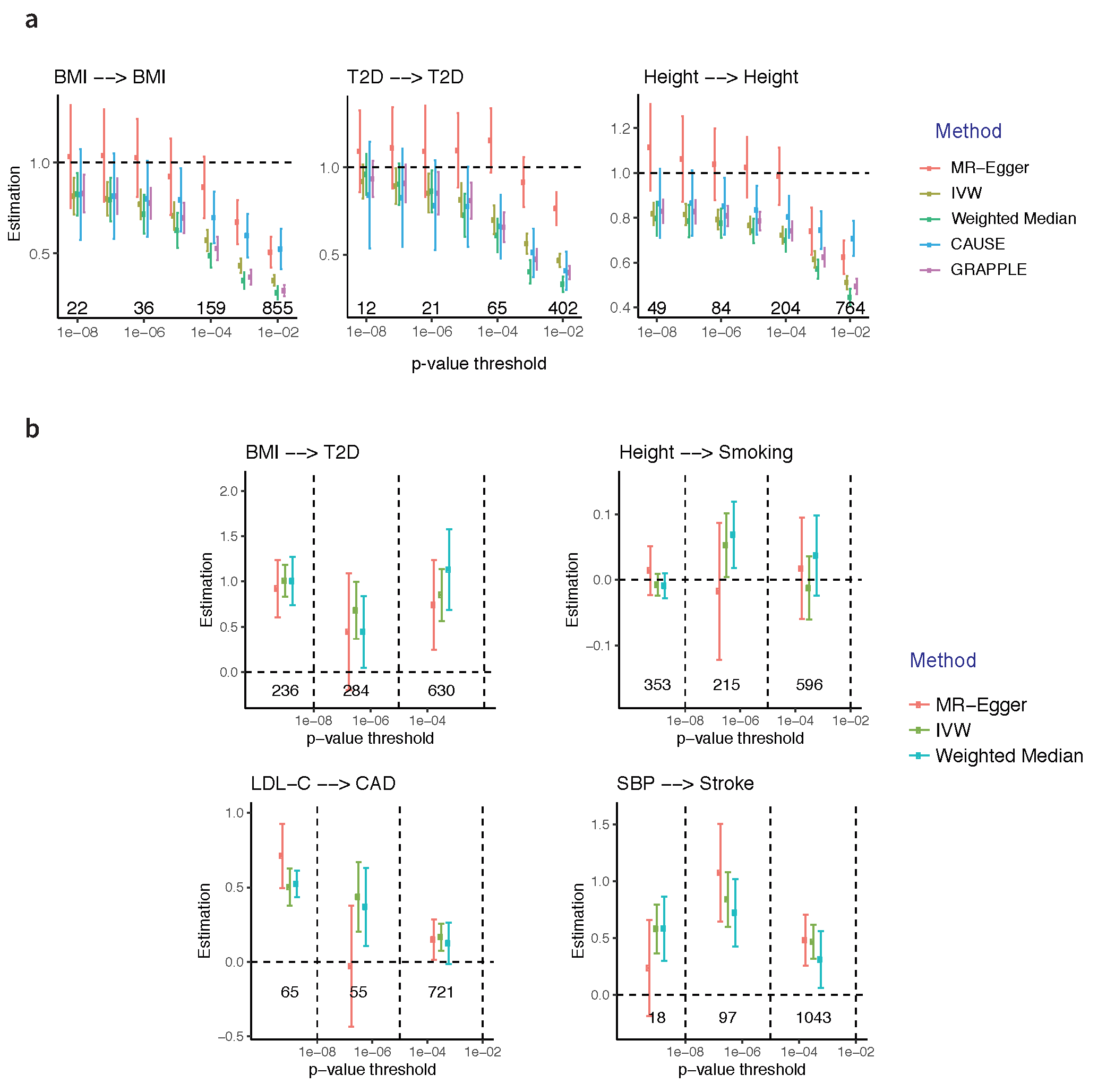

Supplement: S1 Fig — a, Selection bias in MR methods when SNP selection and γ^j are obtained from the same GWAS dataset. True β ≈ 1 and error bars show 95% confidence intervals. The numbers are the number of clumped SNPs at different threshold. b, The estimate of β across three independent categories of SNPs with different association strengths for four risk factor and disease pairs using three other bench-marking MR methods. The numbers are the number of SNPs in each category, separated by the values of their selection p-values (dashed vertical lines). (TIFF) [file pgen.1009575.s001.tiff]

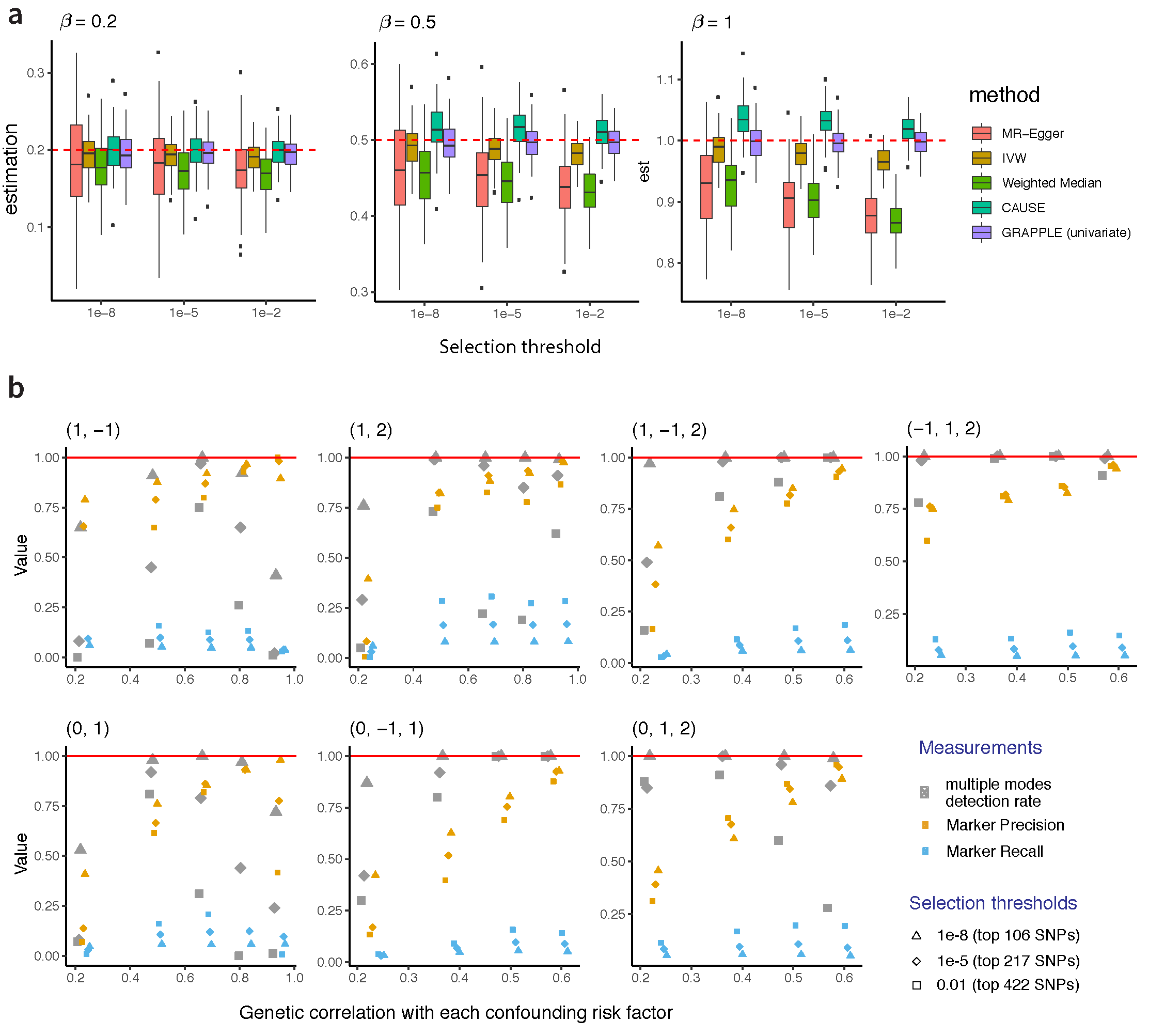

Supplement: S2 Fig — a, Boxplots of the estimated β1 using different MR methods over 100 repeated random experiments when there are no correlated pleiotropy. We compare across three different β1 values (0.2, 0.5 and 1) with SNPs selected by three different selection thresholds: 10−8 for the top 106 SNPs, 10−5 for the top 217 SNPs and 0.01 for the top 422 SNPs. b, Performance of GRAPPLE in detecting multi-modality. In each setting with pleiotropic pathways, we evaluate three metrics: the detection rate of multi-modality, the precision of the identified marker genes of the pleiotropic pathways and the recall of true marker genes that are identified. Each color represent a different metric and each shape is for a different selection threshold. The title of each plot shows (β1, ⋯, βK) in each setting where β1 is the true causal effect, and in each setting, we vary the genetic correlation between each genetic confounding risk factor and the risk factor of interest. (TIFF) [file pgen.1009575.s002.tiff]

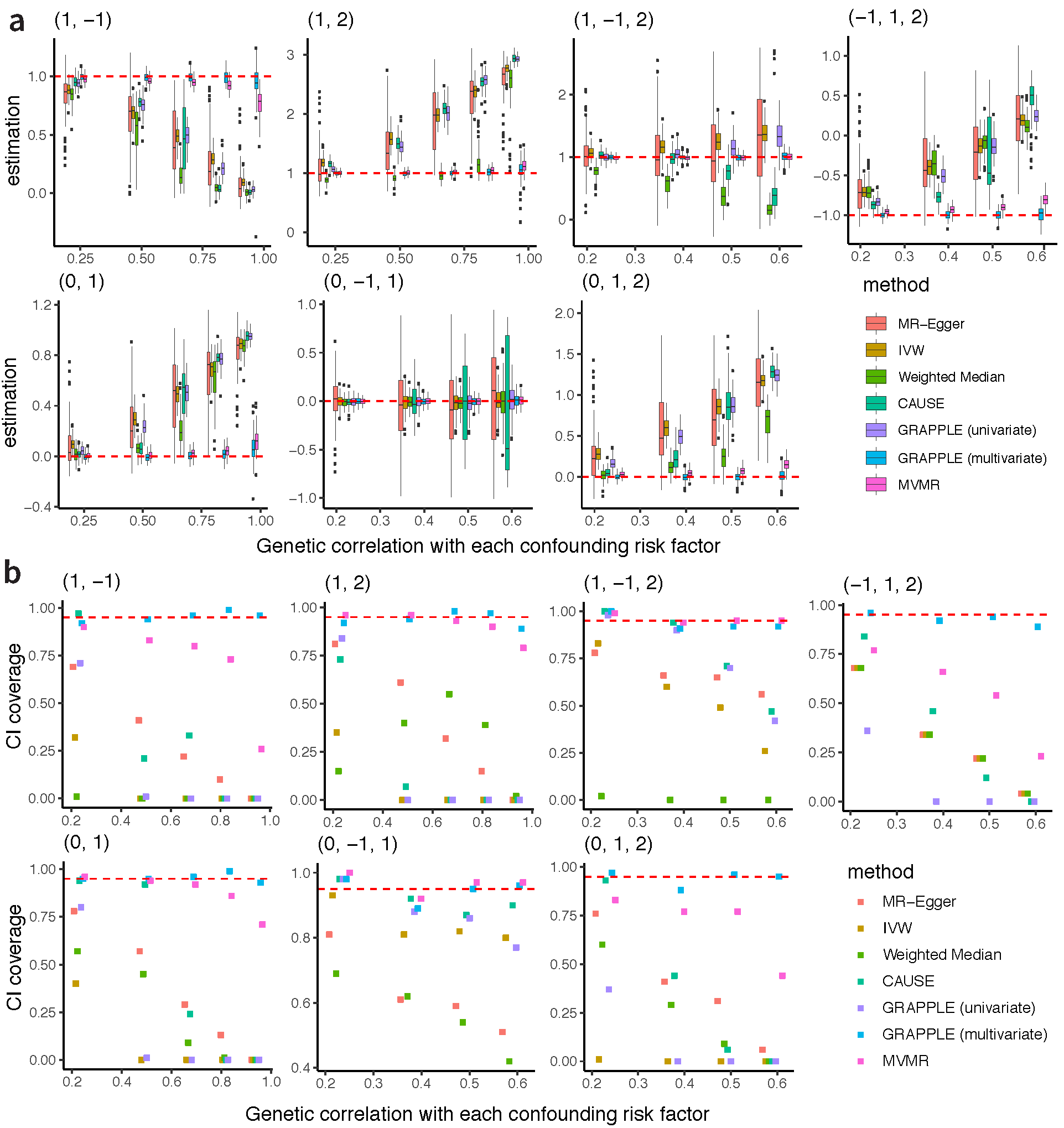

Supplement: S3 Fig — a, Boxplots of the estimated β1 using different MR methods over 100 repeated random experiments. b The actual coverage of the 95% confidence intervals of β1 provided by different methods. For CAUSE, we report the coverage of the 95% credible intervals of β1. The red dotted line shows the expected 0.95 nominal level. For the three settings in the second row with β1 = 0, the CI coverage is the same as 1−type I error. (TIFF) [file pgen.1009575.s003.tiff]

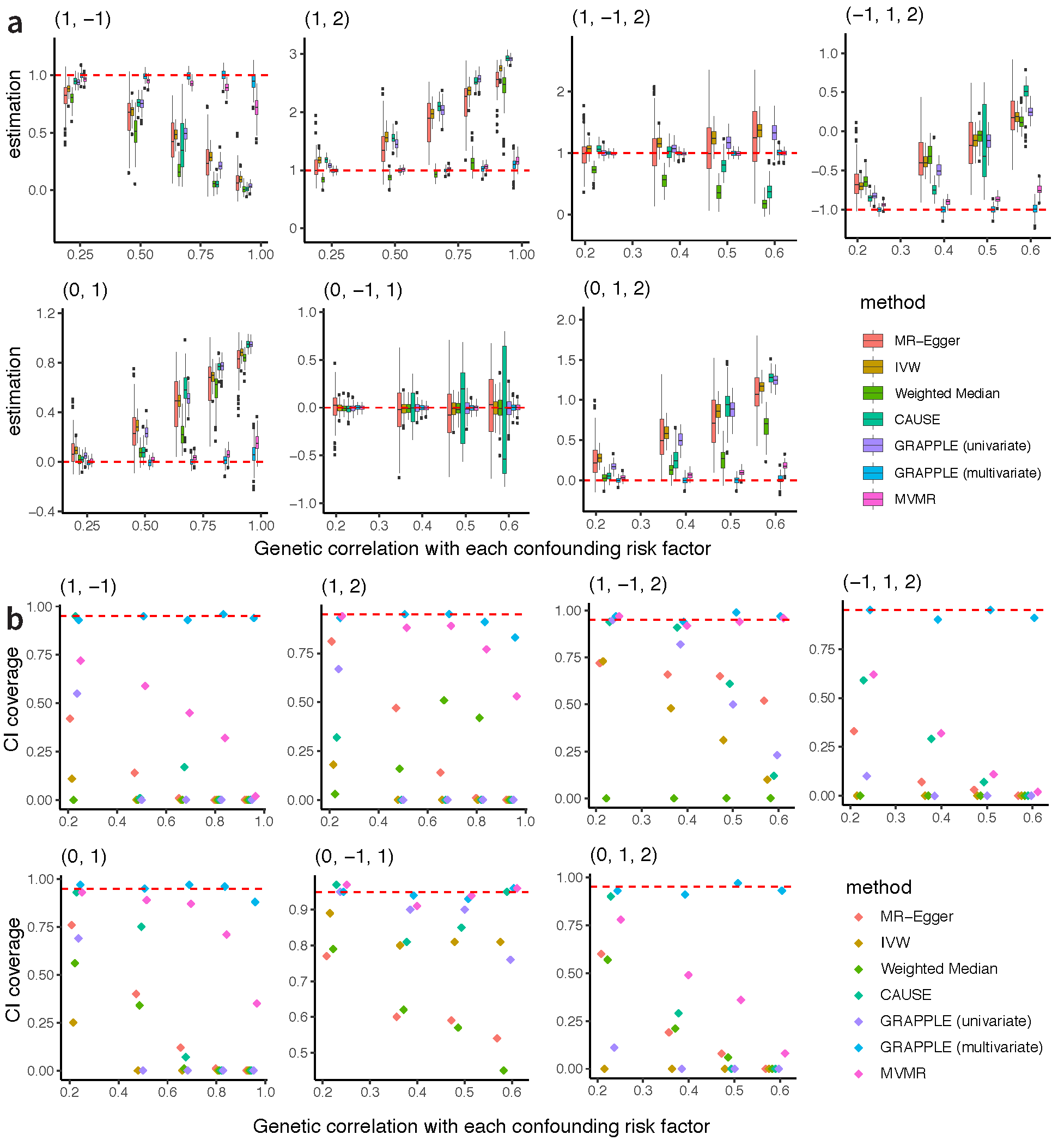

Supplement: S4 Fig — (TIFF) [file pgen.1009575.s004.tiff]

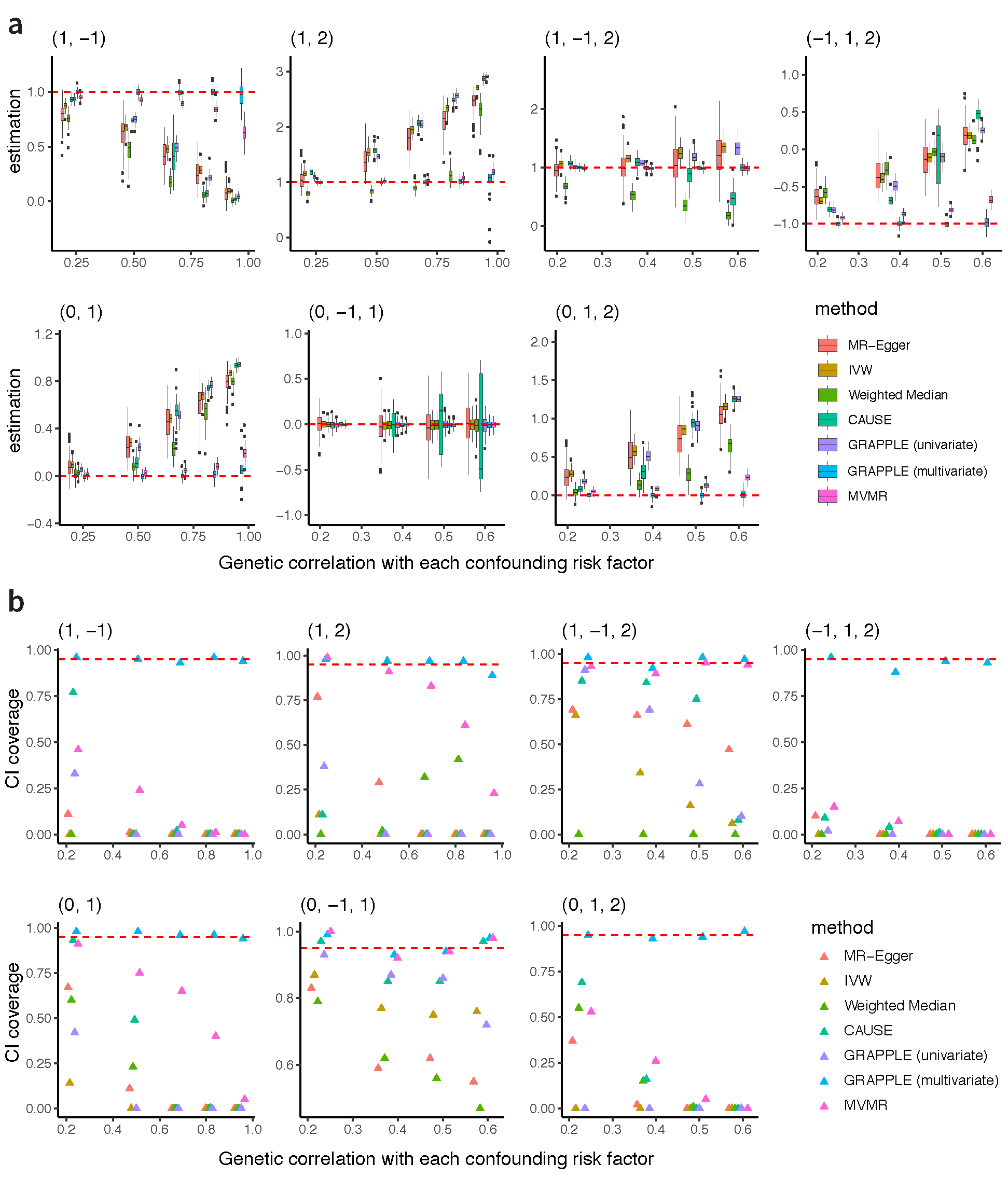

Supplement: S5 Fig — (TIFF) [file pgen.1009575.s005.tiff]

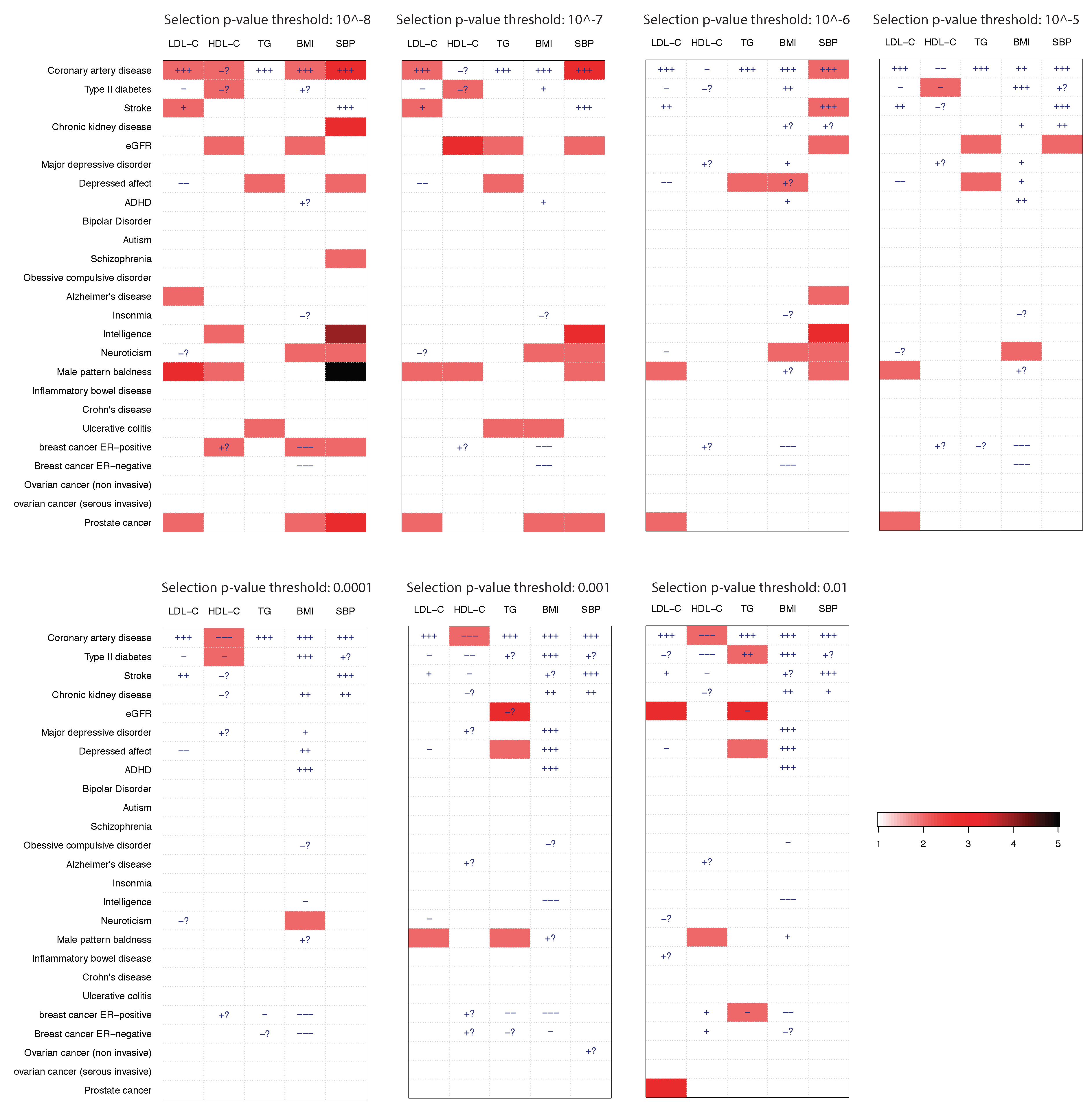

Supplement: S6 Fig — Each figure is for results obtained using one of the 7 p-value thresholds. The colors show the number of detected modes. The “+” sign shows a positive estimated effect and “−” sign shows a negative estimated effect. (TIFF) [file pgen.1009575.s006.tiff]
