# Supplementary material for: Causal inference for heritable phenotypic risk factors using heterogeneous genetic instruments
Source: PLoS Genet. 2021 Jun 22;17(6):e1009575. doi: 10.1371/journal.pgen.1009575 (PMC8301661; doi:10.1371/journal.pgen.1009575)
Supplement: S1 Text — (PDF) [file pgen.1009575.s007.pdf]

S1 Text: Simulations

The simulation model

Our simulation starts with the equation for each SNP j

$$\Gamma_j = \gamma_{j1}\beta_1 + \sum_{k=2}^{K} \gamma_{jk}\beta_k + \alpha_j$$

where we are interested in estimating β_1, the causal effect of our target risk factor X_1. We assume that $\alpha_j \overset{i.i.d.}{\sim} N(0, \tau^2)$ following the inSIDE assumption, while γ_{jk} are correlated with γ_{j1} for $k = 2, 3, \cdots, K$ due to the genetic correlations between X_1 and the confounding unmeasured risk factors $X_2, \cdots, X_K$. Specifically, we assume that for each $k = 2, 3, \cdots, K$,

$$\gamma_{jk} = \eta_{jk}\gamma_{j1} + (1 - \eta_{jk})\delta_{jk}$$

where

$$\eta_{jk} \overset{i.i.d.}{\sim} \text{Bernoulli}(\pi_k), \quad \delta_{jk} \overset{i.i.d.}{\sim} N(0, \tau_k^2)$$

and the data are the summary statistics $\hat{\Gamma}_j$ and $\hat{\gamma}_{1j}$ where

$$\hat{\Gamma}_j \overset{ind.}{\sim} N(\Gamma_j, \sigma_{Yj}^2), \quad \hat{\gamma}_{j1} \overset{ind.}{\sim} N(\gamma_{j1}, \sigma_{Xj}^2).$$

The simulation settings

We base on real data estimations to set realistic values of γ_{j1} as well as the standard errors σ_{Y_j} and σ_{X_j} in our simulations. Specifically, we take the BMI as the risk factor and SBP as the disease. With three-sample design, the BMI summary statistics from the GIANT consortium are used for SNP selection, and data from the UK Biobank for the two traits are used for estimation (see Table A of S3 Text). With a p-value threshold of 0.01, we selected 786 independent SNPs. We treat the estimated $\widehat{\gamma}_{j1}$ for these SNPs as the true marginal associations γ_{j1}. The standard errors σ_{Y_j} and σ_{X_j} are the same as obtained from the GWAS summary statistics. We set $\tau^2 = \sum_j \gamma_{j1}^2/5$ for size of the uncorrelated pleiotropy and $\tau_2^2 = \cdots = \tau_K^2 = \tau^2/2$ for the SNPs effects on confounding risk factors when the SNPs are not on the shared pleiotropic pathway. Instead of using original selection p-values, we redefine the "selection p-value" as $2[1 - \Phi(|\gamma_{j1}|/\sigma_{Xj})]$ representing the signal strengths. Here $\Phi(\cdot)$ is the cumulative density function of the standard normal distribution.

Given the above settings, different π_k corresponds to a different genetic correlation [1] between X_1 and X_k following:

$$\rho_g(X_1, X_k) = \frac{\pi_k}{\sqrt{\pi_k + (1 - \pi_k)/10}}$$

We examine 10 combinations of $(\beta_1, \cdots, \beta_K)$ where $K = 1$, 2 or 3. When there are pleiotropic pathways, we consider both the scenario that the confounding risk factor has same or opposite sign as the effect of the target risk factor. Specifically, the ten combinations are:

- No pleiotropic pathways with $\beta_1 = 0.2$, 0.5 and 1.
- True causal effect is zero, one pleiotropic pathway: $(\beta_1, \beta_2) = (0, 1)$
- True causal effect is zero, with two pleiotropic pathways: $(\beta_1, \beta_2, \beta_3) = (0, -1, 1)$ or $(0, 1, 2)$;
- True causal effect is nonzero, one pleiotropic pathway: $(\beta_1, \beta_2) = (1, -1)$ or $(1, 2)$;
- True causal effect is nonzero, with two pleiotropic pathways: $(\beta_1, \beta_2, \beta_3) = (1, -1, 2)$ or $(-, 1, 2)$.

For each combination, we vary the values of π_k. When $K = 2$, there is only one pleiotropic pathway, and we set $\pi_2 = 0.1, 0.3, 0.5, 0.7, 0.9$. Here $\pi_2 = 0.1$ indicates that $\rho_g(X_1, X_2)$ is as small as 0.23 and $\pi_2 = 0.9$ is for the genetic correlation to be as large as 0.94. For $K = 3$ where there are two pleiotropic pathways, we require $\eta_{jk} = 1$ in at most one k and we set $\pi_2 = \pi_3 = 0.1, 0.2, 0.3, 0.4$. This means that the two pleiotropic pathways have 0 genetic correlation, while they have the same genetic correlation with X_1, ranging from 0.23 ($\pi_2 = \pi_3 = 0.1$) to 0.59 ($\pi_2 = \pi_3 = 0.4$). For each $\boldsymbol{\beta}$ and $\boldsymbol{\pi}$ combination, we randomly repeat the experiments by $B = 100$ times.

The simulation results

We compare GRAPPLE with 4 different MR methods that only take into account 1 risk factor: MR-Egger, IVW, Weighted Median and CAUSE, as well as a recently proposed MR method MVMR [2] for joint MR analysis of multiple risk factors. In the settings where there are pleiotropic pathways, we compares two methods in GRAPPLE. One is GRAPPLE using only the GWAS summary statistics for the target risk factor, where we use MR-RAPS to estimate the causal effect. The other is to perform a multivariate MR analysis including summary statistics from both the target risk factor and confounding risk factors.

First, when there is no pleiotropic pathway, all MR methods are able to provide a good estimation of the true causal effects (Fig S2a). Similar to want we have observed in the benchmarking results based on real data (Fig 2a), we observe that the common MR methods MR-Egger, IVW and Weighted Median can provide conservatively biased estimation even with a stringent selection threshold. GRAPPLE can keep providing an accurate and unbiased estimate of the causal effect regardless of the selection threshold, with the accuracy increasing with a more relaxed selection threshold. CAUSE may sometimes overestimate the true causal effect, as what we have already seen in Fig 2a.

Next, we examine the power of GRAPPLE in detecting multiple modes and finding marker genes for each mode. We use three measurements. One is the detection rate, which is the chance of detecting more than one mode in the robustified profile likelihood. The other two measures are the marker precision and recall. We compare the marker SNPs that GRAPPLE returns with the set of SNPs that truly belong to the pleiotropic pathways. As shown in Fig S2b, the detection of multiple modes performs the best when π_k is neither too large nor too small, so that each pathway has enough SNPs among the selected IVs to contribute to a mode. When we vary the selection thresholds, we find that GRAPPLE

is most sensitive to detect the modes with a stringent p-value threshold (10^{-8}) where only strongly associated SNPs are selected. On the other hand, including weakly associated SNPs will increase the marker recall rate, making it more informative to identify the hidden risk factors once the modes are detected.

Then, we compare GRAPPLE with other methods when there are pleiotropic pathways (Figs S3-S5), using SNPs selected by different selection thresholds. We use two metrics for evaluation: the accuracy in the estimation of the true causal effect, and the coverage of the 95% confidence intervals from each MR method. For CAUSE, we report the 95% credible interval. When the true causal effect $\beta_1 = 0$, the CI coverage is equivalent to one minus the type-I error in claiming a non-zero causal effect. In terms of the estimation of the true causal effects, we observe that all univariate MR methods using only summary statistics of the target risk factor can have large bias when there are pleiotropic pathways, especially when the confounding unmeasured risk factors are highly or even moderately correlated with the target risk factor. In terms of the CI coverage, all univariate MR methods are generally not reliable when there are pleiotropic pathways and result in a larger type-I error than expected. Among all 5 univariate MR methods, CAUSE has the best coverage when the confounding risk factors has a small genetic correlation with the target risk factor, a scenario where the assumptions in CAUSE are likely to hold. Though our univariate GRAPPLE can sometimes provide a less biased estimation of β_1 compared with other univariate MR methods when there are pleiotropic pathways, our CIs are always too optimistic. Fortunately, GRAPPLE also has high detection rate of multi-modality in these scenarios with reasonably high marker precision, so that we are aware of the existence of pleiotropic pathways and have information to identify them.

Finally, we compare the multivariate GRAPPLE with MVMR, both of which use summary statistics from both the target and confounding risk factors (Figs S3-S5). Compared to the univariate MR methods, both methods are much more accurate in estimation and CI coverage. MVMR can suffer from weak instrument bias and is also sensitive to the genetic correlation between confounding risk factors and the target risk factor. In contrast, our multivariate GRAPPLE can keep providing accurate estimation of β_1, as well as reliable CIs with low type-I error regardless of either the genetic correlations of confounding risk factors or the SNPs selection thresholds.

References

[1] B. Bulik-Sullivan, H. K. Finucane, V. Anttila, A. Gusev, F. R. Day, P.-R. Loh, L. Duncan, J. R. Perry, N. Patterson, E. B. Robinson, et al. An atlas of genetic correlations across human diseases and traits. *Nature genetics*, 47(11):1236, 2015.

[2] E. Sanderson, G. Davey Smith, F. Windmeijer, and J. Bowden. An examination of multivariable mendelian randomization in the single-sample and two-sample summary data settings. *International journal of epidemiology*, 48(3):713–727, 2019.
