# Supplementary material for: Causal inference for heritable phenotypic risk factors using heterogeneous genetic instruments
Source: PLoS Genet. 2021 Jun 22;17(6):e1009575. doi: 10.1371/journal.pgen.1009575 (PMC8301661; doi:10.1371/journal.pgen.1009575)
Supplement: S2 Text — (PDF) [file pgen.1009575.s008.pdf]

S2 Text: Additional mathematical details

Correlations of GWAS summary statistics from overlapping cohorts

We provide detailed calculations for the derivation of Equation (8) in the maintext.:

$$\mathrm{Corr}\left[\widehat{\Gamma}_j, \widehat{\gamma}_{jk}\right] \approx \frac{N_{sk}}{\sqrt{N_{ek}N_o}} \mathrm{Corr}\left[Y_s, X_{ks}\right].$$

For any shared sample s, let

$$Y_s = \Gamma_j Z_{js} + \epsilon_{js}, \quad X_{ks} = \gamma_{jk} Z_{js} + e_{jks}$$

where Y_s, X_{ks} and Z_{js} all have mean 0 and the Z_{js} has variance 1 for convenience. Then as for most SNPs, its individual genetic effect is very small, with $\Gamma_j = o(1)$ and $\gamma_{jk} = o(1)$ we have

$$\mathrm{Cov}\left[Y_s, X_{ks}\right] = \Gamma_j \gamma_{jks} \mathrm{Var}\left[Z_{js}\right] + \mathrm{Cov}\left[\epsilon_{js}, e_{jks}\right] \approx \mathrm{Cov}\left[\epsilon_{js}, e_{jks}\right]$$

The summary statistics are computed from marginal regression, so we have

$$\widehat{\gamma}_j = \frac{\widehat{\mathrm{Cov}}_{n_X}(X, Z_j)}{\widehat{\mathrm{Cov}}_{n_X}(Z_j, Z_j)} = \widehat{\mathrm{Cov}}_{n_X}(X, Z_j),\ \widehat{\Gamma}_j = \frac{\widehat{\mathrm{Cov}}_{n_Y}(Y, Z_j)}{\widehat{\mathrm{Cov}}_{n_Y}(Z_j, Z_j)} = \widehat{\mathrm{Cov}}_{n_Y}(Y, Z_j).$$

where $\widehat{\mathrm{Cov}}_n$ is the sample covariance operator.

Thus,

$$\begin{aligned}
\mathrm{Cov}\left[\widehat{\Gamma}_j, \widehat{\gamma}_{jk}\right] &= \frac{1}{N_{ek}N_o} \mathrm{Cov}\left[\sum_{s=1}^{N_{sk}} Y_s Z_{js}, \sum_{s=1}^{N_{sk}} X_{sk} Z_{js}\right] \\
&= \frac{N_{sk}}{N_{ek}N_o} \mathrm{Cov}\left[Y_s Z_{js}, X_{sk} Z_{js}\right] \\
&= \frac{N_{sk}}{N_{ek}N_o} \left(\Gamma_j \gamma_{jk} \mathrm{Var}\left[Z_{js}^2\right] + \mathrm{Var}\left[Z_{js}\right] \mathrm{Cov}\left[\epsilon_{js}, e_{jks}\right]\right) \\
&\approx \frac{N_{sk}}{N_{ek}N_o} \mathrm{Var}\left[Z_{js}\right] \mathrm{Cov}\left[\epsilon_{js}, e_{jks}\right]
\end{aligned}$$

and

$$\begin{aligned}
\mathrm{Var}\left[\widehat{\Gamma}_j\right] &= \frac{1}{N_o} \mathrm{Var}\left[Y_s Z_{js}\right] \approx \frac{1}{N_o} \mathrm{Var}\left[Z_{js}\right] \mathrm{Var}\left[\epsilon_{js}\right] \\
\mathrm{Var}\left[\widehat{\gamma}_{jk}\right] &= \frac{1}{N_{ek}} \mathrm{Var}\left[Y_s Z_{js}\right] \approx \frac{1}{N_{ek}} \mathrm{Var}\left[Z_{js}\right] \mathrm{Var}\left[e_{jks}\right] \\
\mathrm{Var}\left[Y_s\right] &\approx \mathrm{Var}\left[\epsilon_{js}\right], \quad \mathrm{Var}\left[X_{ks}\right] \approx \mathrm{Var}\left[e_{jks}\right]
\end{aligned}$$

Above approximations show that

$$\mathrm{Corr}\left[\widehat{\Gamma}_j, \widehat{\gamma}_{jk}\right] \approx \frac{N_{sk}}{\sqrt{N_{ek}N_o}} \mathrm{Corr}\left[Y_s, X_{ks}\right]$$

Robustified profile likelihood methods for multivariate MR

With the random effect model on α_j, we further have

$$\begin{pmatrix}\widehat{\Gamma}_j\\ \widehat{\boldsymbol{\gamma}}_j\end{pmatrix} \sim \mathcal{N}\left(\begin{pmatrix}\boldsymbol{\gamma}_j^T\boldsymbol{\beta}\\ \boldsymbol{\gamma}_j\end{pmatrix}, \text{diag}\begin{pmatrix}\sigma_{Y_j}\\ \sigma_{X_j}\end{pmatrix}\Sigma\ \text{diag}\begin{pmatrix}\sigma_{Y_j}\\ \sigma_{X_j}\end{pmatrix} + \begin{pmatrix}\tau^2 & \mathbf{0}\\ \mathbf{0} & \mathbf{0}\end{pmatrix}\right)$$

Then, we estimate the parameters $\boldsymbol{\beta}$ and τ^2 using $K+1$ estimation equations. Recall that we have defined

$$t_j(\boldsymbol{\beta}, \tau^2) = \frac{\widehat{\Gamma}_j - \widehat{\boldsymbol{\gamma}}_j{}^T\boldsymbol{\beta}}{\sqrt{\sigma_{Y_j}^2 + \boldsymbol{\beta}^T\Sigma_{\boldsymbol{X}_j}\boldsymbol{\beta} - 2\boldsymbol{\beta}^T\Sigma_{\boldsymbol{X}_jY_j} + \tau^2}} \tag{1}$$

and the robust profile likelihood is the optimization function

$$l(\boldsymbol{\beta}, \tau^2) = -\sum_j l_j(\boldsymbol{\beta}, \tau^2) = -\sum_j \rho\left(t_j(\boldsymbol{\beta}, \tau^2)\right) \tag{2}$$

The estimation equations for $\boldsymbol{\beta}$ are from the derivatives of the robust profile likelihood:

$$\boldsymbol{\varphi}_1(\boldsymbol{\beta}, \tau^2) = \frac{\partial l(\boldsymbol{\beta}, \tau^2)}{\partial \boldsymbol{\beta}} = \mathbf{0}$$

The estimation equation for τ^2 is

$$\varphi_2(\boldsymbol{\beta}, \tau^2) = l(\boldsymbol{\beta}, \tau^2) - p\eta = 0$$

where $\eta = \mathbb{E}\left[\rho(Z)\right]$ with $Z \sim \mathcal{N}(0,1)$. Let $\boldsymbol{\varphi}(\cdot) = \begin{pmatrix}\boldsymbol{\varphi}_1(\cdot)\\ \varphi_2(\cdot)\end{pmatrix}$.

We use delta method to calculate the asymptotic distribution of $\widehat{\boldsymbol{\beta}}$ and $\widehat{\tau}^2$ obtained from the above estimation equations. To make it clear, we denote the true values of $\boldsymbol{\beta}$ and τ as $\boldsymbol{\beta}_0$ and τ_0. Then, we have

$$\mathbf{0} = \boldsymbol{\varphi}(\widehat{\boldsymbol{\beta}}, \widehat{\tau}^2) \approx \boldsymbol{\varphi}(\boldsymbol{\beta}_0, \tau_0^2) + \dot{\boldsymbol{\varphi}}(\boldsymbol{\beta}_0, \tau_0^2)\begin{pmatrix}\widehat{\boldsymbol{\beta}} - \boldsymbol{\beta}_0\\ \widehat{\tau}_0^2 - \tau_0^2\end{pmatrix}$$

Let

$$A = \mathbb{E}\left[-\dot{\boldsymbol{\varphi}}(\boldsymbol{\beta}_0, \tau_0^2)\right],\ B = \text{Var}\left[\boldsymbol{\varphi}(\boldsymbol{\beta}_0, \tau_0^2)\right]$$

Then if $\widehat{\boldsymbol{\beta}}$ and $\widehat{\tau}^2$ are consistent estimates, we would have asymptotically

$$\begin{pmatrix}\widehat{\boldsymbol{\beta}} - \boldsymbol{\beta}_0\\ \widehat{\tau}_0^2 - \tau_0^2\end{pmatrix} \sim \mathcal{N}(0, A^{-1}BA^{-T})$$

Thus, we only need to estimate A and B.

Let's first discuss how to estimate B. Notice that

$$\boldsymbol{\varphi}_{1j}(\boldsymbol{\beta}, \tau^2) = \rho'(t_j)\frac{\partial t_j}{\partial \boldsymbol{\beta}}.$$

Based on the definition of t_j in (1), we have

$$\frac{\partial t_j}{\partial \boldsymbol{\beta}} = -\frac{(\sigma_{Y_j}^2 + \beta^T \Sigma_{X_j} \boldsymbol{\beta} - 2\boldsymbol{\beta}^T \Sigma_{X_j Y_j} + \tau^2)\widehat{\boldsymbol{\gamma}}_j + (\widehat{\Gamma}_j - \widehat{\boldsymbol{\gamma}}_j{}^T \boldsymbol{\beta})(\Sigma_{X_j}\boldsymbol{\beta} - \Sigma_{X_j Y_j})}{\left(\sigma_{Y_j}^2 + \boldsymbol{\beta}^T \Sigma_{X_j} \boldsymbol{\beta} - 2\boldsymbol{\beta}^T \Sigma_{X_j Y_j} + \tau^2\right)^{3/2}}$$

As

$$\operatorname{Var}\left(\widehat{\Gamma}_j - \widehat{\boldsymbol{\gamma}}_j{}^T \boldsymbol{\beta}_0\right) = \sigma_{Y_j}^2 + \boldsymbol{\beta}_0{}^T \Sigma_{X_j} \boldsymbol{\beta}_0 - 2\boldsymbol{\beta}^T \Sigma_{X_j Y_j} + \tau_0^2$$

and

$$\operatorname{Cov}\left(\widehat{\boldsymbol{\gamma}}_j, \widehat{\Gamma}_j - \widehat{\boldsymbol{\gamma}}_j{}^T \boldsymbol{\beta}_0\right) = \Sigma_{X_j Y_j} - \Sigma_{X_j} \boldsymbol{\beta}_0$$

We have,

$$\operatorname{Cov}\left(t_j, \frac{\partial t_j}{\partial \boldsymbol{\beta}}\right) = 0$$

at the true values $\boldsymbol{\beta}_0$ and τ_0. Further because t_j and $\frac{\partial t_j}{\partial \boldsymbol{\beta}}$ are linear transformations of $\widehat{\Gamma}_j$ and $\widehat{\boldsymbol{\gamma}}_j$, they are jointly Gaussian. Thus, t_j and $\frac{\partial t_j}{\partial \boldsymbol{\beta}}$ are independent at the true values $\boldsymbol{\beta}_0$ and τ_0. So we have

$$\begin{aligned}
\operatorname{Var}\left[\boldsymbol{\varphi}_1(\boldsymbol{\beta}_0, \tau_0^2)\right] &= \sum_j \operatorname{Var}\left[\boldsymbol{\varphi}_{1j}(\boldsymbol{\beta}_0, \tau_0^2)\right] \\
&= \sum_j \mathbb{E}\left[\boldsymbol{\varphi}_{1j}(\boldsymbol{\beta}_0, \tau_0^2)^2\right] \\
&= \sum_j \mathbb{E}\left[\rho'(t_j(\boldsymbol{\beta}_0, \tau_0^2))^2\right] \mathbb{E}\left[\frac{\partial t_j}{\partial \boldsymbol{\beta}}(\boldsymbol{\beta}_0, \tau_0^2) \frac{\partial t_j}{\partial \boldsymbol{\beta}}(\boldsymbol{\beta}_0, \tau_0^2)^T\right] \\
&= \mathbb{E}\left[\rho'(Z)^2\right] \sum_j \mathbb{E}\left[\frac{\partial t_j}{\partial \boldsymbol{\beta}}(\boldsymbol{\beta}_0, \tau_0^2) \frac{\partial t_j}{\partial \boldsymbol{\beta}}(\boldsymbol{\beta}_0, \tau_0^2)^T\right]
\end{aligned}$$

Also, we have

$$\operatorname{Cov}\left[\boldsymbol{\varphi}_{1j}(\boldsymbol{\beta}_0, \tau_0^2), \varphi_{2j}(\boldsymbol{\beta}_0, \tau_0^2)\right] = \sum_j \mathbb{E}\left[\rho'(t_j(\boldsymbol{\beta}_0, \tau_0^2)) \frac{\partial t_j}{\partial \boldsymbol{\beta}}(\boldsymbol{\beta}_0, \tau_0^2) \rho'(t_j(\boldsymbol{\beta}_0, \tau_0^2))\right] = 0$$

and

$$\operatorname{Var}\left[\varphi_{2j}(\boldsymbol{\beta}_0, \tau_0^2)\right] = p \operatorname{Var}\left[\rho(Z)\right]$$

where $Z \sim N(0, 1)$. Thus, we can estimate B as

$$\widehat{B} = \begin{pmatrix} \operatorname{Var}\left[\rho(Z)\right] \sum_j \frac{\partial t_j}{\partial \boldsymbol{\beta}}(\widehat{\boldsymbol{\beta}}, \widehat{\tau}^2) \frac{\partial t_j}{\partial \boldsymbol{\beta}}(\widehat{\boldsymbol{\beta}}, \widehat{\tau}^2)^T & 0 \\ 0 & p \operatorname{Var}\left[\rho(Z)\right] \end{pmatrix}$$

Next, we estimate A. We need to calculate the function of $\dot{\boldsymbol{\varphi}}(\boldsymbol{\beta}, \tau^2)$. we have

$$\begin{aligned}
\frac{\partial \boldsymbol{\varphi}_{1j}}{\partial \boldsymbol{\beta}} &= \rho''(t_j)\left(\frac{\partial t_j}{\partial \boldsymbol{\beta}}\right)\left(\frac{\partial t_j}{\partial \boldsymbol{\beta}}\right)^T + \rho'(t_j)\frac{\partial^2 t_j}{\partial \boldsymbol{\beta}\partial \boldsymbol{\beta}} \\
\frac{\partial \boldsymbol{\varphi}_{1j}}{\partial \tau^2} &= \rho''(t_j)\left(\frac{\partial t_j}{\partial \tau^2}\right)\left(\frac{\partial t_j}{\partial \boldsymbol{\beta}}\right) + \rho'(t_j)\frac{\partial^2 t_j}{\partial \tau^2\partial \boldsymbol{\beta}} \\
\frac{\partial \varphi_{2j}}{\partial \boldsymbol{\beta}} &= \boldsymbol{\varphi}_{1j} \\
\frac{\partial \varphi_{2j}}{\partial \tau^2} &= \rho'(t_j)\frac{\partial t_j}{\partial \tau^2}
\end{aligned}$$

As

$$\frac{\partial t_j}{\partial \tau^2} = -\frac{1}{2}\frac{t_j}{\sigma_{Y_j}^2 + \boldsymbol{\beta}^T\Sigma_{X_j}\boldsymbol{\beta} - 2\boldsymbol{\beta}^T\Sigma_{X_jY_j} + \tau^2}$$

we have

$$\mathbb{E}\left[\frac{\partial \varphi_{2j}}{\partial \tau^2}(\boldsymbol{\beta}_0, \tau_0^2)\right] = -\frac{\mathbb{E}\left[\rho'(Z)Z\right]}{2}\sum_j \frac{1}{\sigma_{Y_j}^2 + \boldsymbol{\beta}_0^T\Sigma_{X_j}\boldsymbol{\beta}_0 - 2\boldsymbol{\beta}_0^T\Sigma_{X_jY_j} + \tau_0^2}$$

$$\begin{aligned}
\mathbb{E}\left[\frac{\partial \boldsymbol{\varphi}_{1j}}{\partial \tau^2}(\boldsymbol{\beta}_0, \tau_0^2)\right] &= \mathbb{E}\left[\rho'(t_j(\boldsymbol{\beta}_0, \tau_0^2))\frac{\partial^2 t_j}{\partial \tau^2\partial \boldsymbol{\beta}}(\boldsymbol{\beta}_0, \tau_0^2)\right] \\
&= \frac{\mathbb{E}\left[\rho'(Z)Z\right]}{2}\sum_j \frac{\Sigma_{X_j}\boldsymbol{\beta} - \Sigma_{X_jY_j}}{(\sigma_{Y_j}^2 + \boldsymbol{\beta}_0^T\Sigma_{X_j}\boldsymbol{\beta}_0 - 2\boldsymbol{\beta}_0^T\Sigma_{X_jY_j} + \tau_0^2)^2}
\end{aligned}$$

We also have

$$\mathbb{E}\left[\frac{\partial \varphi_{2j}}{\partial \boldsymbol{\beta}}(\boldsymbol{\beta}_0, \tau_0^2)\right] = \mathbf{0}$$

$$\begin{aligned}
\mathbb{E}\left[\frac{\partial \boldsymbol{\varphi}_{1j}}{\partial \boldsymbol{\beta}}(\boldsymbol{\beta}_0, \tau_0^2)\right] =& \mathbb{E}\left[\rho''(Z)\right]\mathbb{E}\left[\sum_j \frac{\partial t_j}{\partial \boldsymbol{\beta}}(\boldsymbol{\beta}_0, \tau_0^2)\frac{\partial t_j}{\partial \boldsymbol{\beta}}(\boldsymbol{\beta}_0, \tau_0^2)^T\right] + \\
& \mathbb{E}\left[\sum_j \rho'(t_j(\boldsymbol{\beta}_0, \tau_0^2))\frac{\partial^2 t_j}{\partial \boldsymbol{\beta}\partial \boldsymbol{\beta}}(\boldsymbol{\beta}_0, \tau_0^2)\right]
\end{aligned}$$

where we can estimate the last two expectations on the right hand side by the corresponding sample means.
