# Supplementary material for: Causal inference for heritable phenotypic risk factors using heterogeneous genetic instruments
Source: PLoS Genet. 2021 Jun 22;17(6):e1009575. doi: 10.1371/journal.pgen.1009575 (PMC8301661; doi:10.1371/journal.pgen.1009575)
Supplement: S3 Text — (PDF) [file pgen.1009575.s009.pdf]

S3 Text: Data resources

We list the datasets that we have used in the paper and their download sources. Table A and table B summarize how these datasets are used in different analyses in the paper. Intermediate results for screening of 5 risk factors on 25 diseases are available at https://www.dropbox.com/sh/myh8xgxne8fo17v/AABWJf781VrCGnqNFMLtnqIea?dl=0.

Analysis	Risk factor datasets		Disease dataset
	Selection	Estimation	
Validation1:			
BMI → BMI	BMI-ukb	BMI-giant17eu-F	BMI-giant17eu-M
T2D → T2D	T2D-ukb	T2D-diagram12-F	T2D-diagram12-M
Height → Height	Height-ukb	Height-giant13-F	Height-giant13-M
Validation2:			
BMI → T2D	BMI-ukb	BMI-giant17eu	T2D-diagram12-Im
LDL-C → CAD	LDL-gera18	LDL-glgc13	CAD-Nelson17
Height → Smoking	Height-giant14	Height-ukb	Smoking-ukb19
SBP → Stroke	SBP-gera17	SBP-ukb	AS-Malik18eu
Validation3:			
BMI → T2D	BMI-ukb	BMI-giant17eu-F/M	T2D-diagram-F/M
T2D → BMI	T2D-ukb	T2D-diagram12-F/M	BMI-giant17eu-F/M
LDL-C → CAD	LDL-gera18	LDL-glgc13	CAD-Nelson17
CAD → LDL-C	CAD-c4d11	CAD-CARDIoGRAM11	LDL-glgc13
Validation4:			
CRP → CAD	CRP-Prins17	CRP-Dehghan11	CAD-Nelson17
CRP + LDL-C → CAD	(CRP-Prins17, LDL-gera18)	(CRP-Dehghan11, LDL-glgc13)	CAD-Nelson17
Screening risk factors:			
LDL-C	LDL-gera18	LDL-glgc13	-
HDL-C	HDL-gera18	HDL-glgc13	-
TG-C	TG-gera18	TG-glgc13	-
BMI	BMI-JapB	BMI-ukb	-
SBP	SBP-gera17	SBP-ukb	-
Simulation:			
BMI → SBP	BMI-giant17eu	BMI-ukb	SBP-ukb

Table A: Names of the datasets used in validation analyses and screening risk factors

- BMI-ukb: downloaded from UK Biobank Neale's lab [12] with phenotype code 21001.

Screening Disease	dataset	Screening Disease	dataset
CAD	CAD-Nelson17	Insomnia	Insomnia-ukb19
Type 2 diabetes	T2D-diagram12-Im	Intelligence	IQ-Savage18
Stroke	AS-Malik18eu	Neuroticism	Neuro-Hill19
Chronic kidney disease	CKD-Wuttke19	Male pattern baldness	MBP-ukb19
eGFR	eGFR-Wuttke19	IBD	IBD-Liu15
MDD	MDD-PGC18	Crohn's disease	CR-Liu15
Depressed affect	Dep-Nagel18	Ulcerative colitis	UC-Liu15
ADHD	ADHD-pgc19	Breast cancer ER+	Breast-Micha7erp
Bipolar Disorder	BIP-pgc19	Breast cancer ER-	Breast-Micha17ern
Autism	Autism-pgc17	Ovarian cancer (non-invasive)	Ovarian-Phelan17ni
Schizophrenia	SCZ-pgc13		
Obsessive compulsive disorder	OCD-pgc18	Ovarian cancer (serous invasive)	Ovarian-Phelan17si
Alzheimer	Alzhe-Marioni18	Prostate cancer	Prostate-ellipse18

Table B: Names of the datasets used for the 25 disesases in the screening application

- BMI-giant17eu-F, BMI-giant17eu-M, BMI-giant17eu: downloaded from GIANT consortium website [2], 2017 adjusted for smoking data.
- BMI-JapB: data originally from [4] and downloaded from GWAS Catalog [3] with study accession number GCST004904.
- T2D-diagram12-F, T2D-diagram12-M, T2D-diagram12-Im: data originally from [19] and downloaded from `https://diagram-consortium.org/downloads.html`.
- T2D-ukb: downloaded from UK Biobank Neale's lab [12] with phenotype code 20002-1223.
- Height-giant13-F, Height-giant13-M: data originally from [24] and downloaded from GIANT consortium website [2].
- Height-giant14: data originally from [31] and downloaded from GIANT consortium website [2].
- Height-ukb: downloaded from UK Biobank Neale's lab [12] with phenotype code 50.
- LDL-glgc13, HDL-glgc13, TG-glgc13: data originally from [30] and downloaded from `http://csg.sph.umich.edu/willer/public/lipids2013/` (Joint analysis of metoboship and GWAS data).
- LDL-gera18, HDL-gera18, TG-gera18: data originally from [11] and downloaded from GWAS Catalog with study accession numbers GCST007141, GCST007140 and GCST007142.
- SBP-gera17: data originally from [10] and downloaded from GWAS Catalog with study accession number GCST007095.

- SBP-ukb: downloaded from UK Biobank Neale's lab [12] with phenotype code 4080.
- CAD-Nelson17: data originally from [21] and downloaded from GWAS Catalog with study accession number GCST004787.
- CAD-c4d11: data originally from [6] and downloaded from http://www.cardiogramplusc4d.org/data-downloads/
- CAD-CARDIoGRAM11: data originally from [28] and downloaded from http://www.cardiogramplusc4d.org/data-downloads/
- CRP-Prins17: data originally from [23] and downloaded from GWAS Catalog with study accession number GCST005067.
- CRP-Dehghan11: Data from [7] and requested from original authors.
- AS-Malik18EU: data originally from [16] and downloaded from GWAS Catalog with study accession number GCST006906.
- Smoking-ukb19: data originally from [14] and downloaded from GWAS Catalog with study accession number GCST007327.
- CKD-Wuttke19, eGFR-Wuttke19: data originally from [33] and downloaded from GWAS Catalog with study accession numbers GCST008065 and GCST008059.
- Dep-Nagel18: data originally from [20] and downloaded from GWAS Catalog with study accession number GCST006475.
- ADHD-pgc19: data originally from [8] and downloaded from the PGC website https://www.med.unc.edu/pgc/download-results/.
- MDD-pgc18: data originally from [32] and downloaded from the PGC website https://www.med.unc.edu/pgc/download-results/.
- BIP-pgc19: data originally from [29] and downloaded from the PGC website https://www.med.unc.edu/pgc/download-results/.
- Autism-pgc17: data originally from [1] and downloaded from the PGC website https://www.med.unc.edu/pgc/download-results/.
- SCZ-pgc13: data originally from [25] and downloaded from the PGC website https://www.med.unc.edu/pgc/download-results/.
- OCD-pgc18: data originally from [5] and downloaded from the PGC website https://www.med.unc.edu/pgc/download-results/.
- Alzhe-Marioni18: data originally from [17] and downloaded from GWAS Catalog with study accession number GCST005922.
- Neuro-Hill19: data originally from [9] and downloaded from GWAS Catalog with study accession number GCST007710.

- IQ-Savage18: data originally from [26] and downloaded from GWAS Catalog with study accession number GCST006250.

- Insomnia-ukb19: data originally from [13] and downloaded from GWAS Catalog with study accession number GCST007387.

- MBP-ukb19: data originally from [34] and downloaded from GWAS Catalog with study accession number GCST007020.

- IBD-Liu15, UC-Liu15, CR-Liu15: data originally from [15] and downloaded from GWAS Catalog with study accession numbers GCST003043, GCST003045 and GCST003044.

- Breast-Micha17erp, Breast-Micha17ern: data originally from [18] and downloaded from GWAS Catalog with study accession number GCST004988.

- Ovarian-Phelan17ni, Ovarian-Phelan17si: data originally from [22] and downloaded from GWAS Catalog with study accession numbers GCST004462 and GCST004478.

- Prostate-ellipse18: data originally from [27] and downloaded from GWAS Catalog with study accession number GCST006085

References

[1] Meta-analysis of gwas of over 16,000 individuals with autism spectrum disorder highlights a novel locus at 10q24. 32 and a significant overlap with schizophrenia. *Molecular autism*, 8:1–17, 2017.

[2] *GIANT consortium data files*, (accessed 2020/3/25). https://portals.broadinstitute.org/collaboration/giant/index.php/GIANT_consortium_data_files.

[3] *GWAS Catalog*, (accessed 2020/3/25). https://www.ebi.ac.uk/gwas/home.

[4] M. Akiyama, Y. Okada, M. Kanai, A. Takahashi, Y. Momozawa, M. Ikeda, N. Iwata, S. Ikegawa, M. Hirata, K. Matsuda, et al. Genome-wide association study identifies 112 new loci for body mass index in the japanese population. *Nature genetics*, 49(10):1458, 2017.

[5] P. D. Arnold, K. D. Askland, C. Barlassina, L. Bellodi, O. Bienvenu, D. Black, M. Bloch, H. Brentani, C. L. Burton, B. Camarena, et al. Revealing the complex genetic architecture of obsessive-compulsive disorder using meta-analysis. *Molecular psychiatry*, 23(5):1181–1181, 2018.

[6] Coronary Artery Disease (C4D) Genetics Consortium et al. A genome-wide association study in europeans and south asians identifies five new loci for coronary artery disease. *Nature genetics*, 43(4):339, 2011.

[7] A. Dehghan, J. Dupuis, M. Barbalic, J. C. Bis, G. Eiriksdottir, C. Lu, N. Pellikka, H. Wallaschofski, J. Kettunen, P. Henneman, et al. Meta-analysis of genome-wide association studies in¿ 80 000 subjects identifies multiple loci for c-reactive protein levelsclinical perspective. *Circulation*, 123(7):731–738, 2011.

[8] D. Demontis, R. K. Walters, J. Martin, M. Mattheisen, T. D. Als, E. Agerbo, G. Baldursson, R. Belliveau, J. Bybjerg-Grauholm, M. Bækvad-Hansen, et al. Discovery of the first genome-wide significant risk loci for attention deficit/hyperactivity disorder. *Nature genetics*, 51(1):63–75, 2019.

[9] W. D. Hill, A. Weiss, D. C. Liewald, G. Davies, D. J. Porteous, C. Hayward, A. M. McIntosh, C. R. Gale, and I. J. Deary. Genetic contributions to two special factors of neuroticism are associated with affluence, higher intelligence, better health, and longer life. *Molecular psychiatry*, pages 1–19, 2019.

[10] T. J. Hoffmann, G. B. Ehret, P. Nandakumar, D. Ranatunga, C. Schaefer, P.-Y. Kwok, C. Iribarren, A. Chakravarti, and N. Risch. Genome-wide association analyses using electronic health records identify new loci influencing blood pressure variation. *Nature genetics*, 49(1):54, 2017.

[11] T. J. Hoffmann, E. Theusch, T. Haldar, D. K. Ranatunga, E. Jorgenson, M. W. Medina, M. N. Kvale, P.-Y. Kwok, C. Schaefer, R. M. Krauss, et al. A large electronic-health-record-based genome-wide study of serum lipids. *Nature genetics*, 50(3):401–413, 2018.

[12] N. lab. *UK Biobank round 1 GWAS results*, 2017 (accessed 2020/3/25). http://www.nealelab.is/blog/2017/7/19/rapid-gwas-of-thousands-of-phenotypes-for-337000-samples-in-the-uk-biobank.

[13] J. M. Lane, S. E. Jones, H. S. Dashti, A. R. Wood, K. G. Aragam, V. T. van Hees, L. B. Strand, B. S. Winsvold, H. Wang, J. Bowden, et al. Biological and clinical insights from genetics of insomnia symptoms. *Nature genetics*, 51(3):387–393, 2019.

[14] R. K. Linnér, P. Biroli, E. Kong, S. F. W. Meddens, R. Wedow, M. A. Fontana, M. Lebreton, S. P. Tino, A. Abdellaoui, A. R. Hammerschlag, et al. Genome-wide association analyses of risk tolerance and risky behaviors in over 1 million individuals identify hundreds of loci and shared genetic influences. *Nature genetics*, 51(2):245–257, 2019.

[15] J. Z. Liu, S. Van Sommeren, H. Huang, S. C. Ng, R. Alberts, A. Takahashi, S. Ripke, J. C. Lee, L. Jostins, T. Shah, et al. Association analyses identify 38 susceptibility loci for inflammatory bowel disease and highlight shared genetic risk across populations. *Nature genetics*, 47(9):979, 2015.

[16] R. Malik, G. Chauhan, M. Traylor, M. Sargurupremraj, Y. Okada, A. Mishra, L. Rutten-Jacobs, A.-K. Giese, S. W. Van Der Laan, S. Gretarsdottir, et al. Multiancestry genome-wide association study of 520,000 subjects identifies 32 loci associated with stroke and stroke subtypes. *Nature genetics*, 50(4):524–537, 2018.

[17] R. E. Marioni, S. E. Harris, Q. Zhang, A. F. McRae, S. P. Hagenaars, W. D. Hill, G. Davies, C. W. Ritchie, C. R. Gale, J. M. Starr, et al. Gwas on family history of alzheimer's disease. *Translational psychiatry*, 8(1):1–7, 2018.

[18] K. Michailidou, S. Lindström, J. Dennis, J. Beesley, S. Hui, S. Kar, A. Lemaçon, P. Soucy, D. Glubb, A. Rostamianfar, et al. Association analysis identifies 65 new breast cancer risk loci. *Nature*, 551(7678):92, 2017.

[19] A. P. Morris, B. F. Voight, T. M. Teslovich, T. Ferreira, A. V. Segre, V. Steinthorsdottir, R. J. Strawbridge, H. Khan, H. Grallert, A. Mahajan, et al. Large-scale association analysis provides insights into the genetic architecture and pathophysiology of type 2 diabetes. *Nature genetics*, 44(9):981, 2012.

[20] M. Nagel, P. R. Jansen, S. Stringer, K. Watanabe, C. A. de Leeuw, J. Bryois, J. E. Savage, A. R. Hammerschlag, N. G. Skene, A. B. Muñoz-Manchado, et al. Meta-analysis of genome-wide association studies for neuroticism in 449,484 individuals identifies novel genetic loci and pathways. *Nature genetics*, 50(7):920–927, 2018.

[21] C. P. Nelson, A. Goel, A. S. Butterworth, S. Kanoni, T. R. Webb, E. Marouli, L. Zeng, I. Ntalla, F. Y. Lai, J. C. Hopewell, et al. Association analyses based on false discovery rate implicate new loci for coronary artery disease. *Nature genetics*, 49(9):1385, 2017.

[22] C. M. Phelan, K. B. Kuchenbaecker, J. P. Tyrer, S. P. Kar, K. Lawrenson, S. J. Winham, J. Dennis, A. Pirie, M. J. Riggan, G. Chornokur, et al. Identification of 12 new susceptibility loci for different histotypes of epithelial ovarian cancer. *Nature genetics*, 49(5):680, 2017.

[23] B. P. Prins, K. B. Kuchenbaecker, Y. Bao, M. Smart, D. Zabaneh, G. Fatemifar, J. Luan, N. J. Wareham, R. A. Scott, J. R. Perry, et al. Genome-wide analysis of health-related biomarkers in the uk household longitudinal study reveals novel associations. *Scientific reports*, 7(1):1–9, 2017.

[24] J. C. Randall, T. W. Winkler, Z. Kutalik, S. I. Berndt, A. U. Jackson, K. L. Monda, T. O. Kilpeläinen, T. Esko, R. Mägi, S. Li, et al. Sex-stratified genome-wide association studies including 270,000 individuals show sexual dimorphism in genetic loci for anthropometric traits. *PLoS Genet*, 9(6):e1003500, 2013.

[25] S. Ripke, C. O'Dushlaine, K. Chambert, J. L. Moran, A. K. Kähler, S. Akterin, S. E. Bergen, A. L. Collins, J. J. Crowley, M. Fromer, et al. Genome-wide association analysis identifies 13 new risk loci for schizophrenia. *Nature genetics*, 45(10):1150, 2013.

[26] J. E. Savage, P. R. Jansen, S. Stringer, K. Watanabe, J. Bryois, C. A. De Leeuw, M. Nagel, S. Awasthi, P. B. Barr, J. R. Coleman, et al. Genome-wide association meta-analysis in 269,867 individuals identifies new genetic and functional links to intelligence. *Nature genetics*, 50(7):912–919, 2018.

[27] F. R. Schumacher, A. A. Al Olama, S. I. Berndt, S. Benlloch, M. Ahmed, E. J. Saunders, T. Dadaev, D. Leongamornlert, E. Anokian, C. Cieza-Borrella, et al. Association

analyses of more than 140,000 men identify 63 new prostate cancer susceptibility loci. *Nature genetics*, 50(7):928, 2018.

[28] H. Schunkert, I. R. König, S. Kathiresan, M. P. Reilly, T. L. Assimes, H. Holm, M. Preuss, A. F. Stewart, M. Barbalic, C. Gieger, et al. Large-scale association analysis identifies 13 new susceptibility loci for coronary artery disease. *Nature genetics*, 43(4):333–338, 2011.

[29] E. A. Stahl, G. Breen, A. J. Forstner, A. McQuillin, S. Ripke, V. Trubetskoy, M. Mattheisen, Y. Wang, J. R. Coleman, H. A. Gaspar, et al. Genome-wide association study identifies 30 loci associated with bipolar disorder. *Nature genetics*, 51(5):793–803, 2019.

[30] C. J. Willer, E. M. Schmidt, S. Sengupta, G. M. Peloso, S. Gustafsson, S. Kanoni, A. Ganna, J. Chen, M. L. Buchkovich, S. Mora, et al. Discovery and refinement of loci associated with lipid levels. *Nature genetics*, 45(11):1274, 2013.

[31] A. R. Wood, T. Esko, J. Yang, S. Vedantam, T. H. Pers, S. Gustafsson, A. Y. Chu, K. Estrada, Z. Kutalik, N. Amin, et al. Defining the role of common variation in the genomic and biological architecture of adult human height. *Nature genetics*, 46(11):1173, 2014.

[32] N. R. Wray, S. Ripke, M. Mattheisen, M. Trzaskowski, E. M. Byrne, A. Abdellaoui, M. J. Adams, E. Agerbo, T. M. Air, T. M. Andlauer, et al. Genome-wide association analyses identify 44 risk variants and refine the genetic architecture of major depression. *Nature genetics*, 50(5):668, 2018.

[33] M. Wuttke, Y. Li, M. Li, K. B. Sieber, M. F. Feitosa, M. Gorski, A. Tin, L. Wang, A. Y. Chu, A. Hoppmann, et al. A catalog of genetic loci associated with kidney function from analyses of a million individuals. *Nature genetics*, 51(6):957, 2019.

[34] C. X. Yap, J. Sidorenko, Y. Wu, K. E. Kemper, J. Yang, N. R. Wray, M. R. Robinson, and P. M. Visscher. Dissection of genetic variation and evidence for pleiotropy in male pattern baldness. *Nature communications*, 9(1):1–12, 2018.
